# Population genomics of diarrheagenic *Escherichia coli* uncovers high connectivity between urban and rural communities in Ecuador

**DOI:** 10.1016/j.meegid.2023.105476

**Published:** 2023-06-29

**Authors:** Andrew P. Rothstein, Kelsey J. Jesser, Dorian J. Feistel, Konstantinos T. Konstantinidis, Gabriel Trueba, Karen Levy

**Affiliations:** a Department of Environmental and Occupational Health Sciences, School of Public Health, University of Washington, Seattle, WA, USA; b School of Civil and Environmental Engineering, Georgia Institute of Technology, Atlanta, GA, USA; c School of a Biological Sciences, Georgia Institute of Technology, Atlanta, GA, USA; d Instituto de Microbiología, Colegio de Ciencias Biológicas y Ambientales, Universidad San Francisco de Quito, Quito, Pichincha, Ecuador

**Keywords:** Diarrheagenic *Escherichia coli*, Population genomics, Genomic epidemiology, Public health, Ecuador, Travel, Diarrhea

## Abstract

Human movement may be an important driver of transmission dynamics for enteric pathogens but has largely been underappreciated except for international ‘travelers’ diarrhea or cholera. Phylodynamic methods, which combine genomic and epidemiological data, are used to examine rates and dynamics of disease matching underlying evolutionary history and biogeographic distributions, but these methods often are not applied to enteric bacterial pathogens. We used phylodynamics to explore the phylogeographic and evolutionary patterns of diarrheagenic *E. coli* in northern Ecuador to investigate the role of human travel in the geographic distribution of strains across the country. Using whole genome sequences of diarrheagenic *E. coli* isolates, we built a core genome phylogeny, reconstructed discrete ancestral states across urban and rural sites, and estimated migration rates between *E. coli* populations. We found minimal structuring based on site locations, urban vs. rural locality, pathotype, or clinical status. Ancestral states of phylogenomic nodes and tips were inferred to have 51% urban ancestry and 49% rural ancestry. Lack of structuring by location or pathotype *E. coli* isolates imply highly connected communities and extensive sharing of genomic characteristics across isolates. Using an approximate structured coalescent model, we estimated rates of migration among circulating isolates were 6.7 times larger for urban towards rural populations compared to rural towards urban populations. This suggests increased inferred migration rates of diarrheagenic *E. coli* from urban populations towards rural populations. Our results indicate that investments in water and sanitation prevention in urban areas could limit the spread of enteric bacterial pathogens among rural populations.

## 1. Introduction

Human movement is known to impact the epidemiology of pathogens (Balcan et al., 2009; Blackburn et al., 2019; Dougherty et al., 2018; Funk et al., 2010; Greenbaum et al., 2019; Meloni et al., 2011; Yang et al., 2011). Increased mobility(Findlater and Bogoch, 2018) and anthropogenic environmental change (Altizer et al., 2013; Gottdenker et al., 2014) have contributed to a rapid expansion (Albery et al., 2020; Carlson et al., 2021; Keim and Wagner, 2009; Lebarbenchon et al., 2008; Patz et al., 2004) and mixing of pathogens into new environments (Dallas et al., 2019; Poisot et al., 2014; K. F. Smith et al., 2007). Consequently, rates of emergence and reemergence of pathogens are expected to increase, with considerable consequences for public health. Numerous studies have highlighted the dramatic impacts of human mobility on disease dynamics including for SARS-CoV-2 (Badr et al., 2020; Chinazzi et al., 2020; Xiong et al., 2020), HIV (Faria et al., 2014; Ratmann et al., 2020; Valdano et al., 2021; Vasylyeva et al., 2018), influenza (Changruenngam et al., 2020; Charu et al., 2017), Dengue (Wesolowski et al., 2015), and antibiotic resistance (Bokhary et al., 2021; I. Frost et al., 2019; Memish et al., 2003).

Urbanization has also increased the ability of pathogens to spread (Alirol et al., 2011; Eskew and Olival, 2018; Neiderud, 2015; Reyes et al., 2013). Rates of urbanization are growing, especially in low- and middle- income countries (LMICs), and both density and connectivity between urban and rural environments can increase disease risks. Examples include Ebola outbreaks attributed to rapid urban expansion (Munster et al., 2018), dengue’s association with urbanization in tropical and subtropical regions (Dutta et al., 1998; Qi et al., 2015; Ren et al., 2019; Wu et al., 2009; Zhu et al., 2016), and measles outbreaks associated with infrastructural connectivity from urban areas (Bharti et al., 2010). We previously found that areas closer to roads in northern Ecuador experience higher risk of diarrheal infections and antibiotic resistance gene carriage than more remote communities (Eisenberg et al., 2006, 2012a).

Despite human movement’s importance in driving pathogen dynamics, it has been underappreciated in enteric disease transmission, apart from studies of traveler’s diarrhea (Shah et al., 2009; Steffen et al., 2004) and cholera (Eppinger et al., 2014; Frerichs et al., 2012; Hendriksen et al., 2011; Phelps et al., 2018; Sack et al., 2021). Diarrheal diseases are a leading contributor to global disease burdens and disproportionately affect children under five in LMICs (Troeger et al., 2017). In addition to causing acute diarrhea, enteric infections can lead to long term sequelae, including environmental enteric dysfunction, growth faltering, and cognitive impairment (Bartelt et al., 2019; Campbell et al., 2003; Goto et al., 2009; Guerrant et al., 2008, 2013; Humphrey, 2009; P. Lunn et al., 1991; P. G. Lunn, 2002; Salameh et al., 2019), contributing to educational and financial achievement gaps in highly affected areas (Adair et al., 2013; Kinyoki et al., 2020; Leroy and Frongillo, 2019).

Water, sanitation, and hygiene (WASH) conditions are primary determinants of diarrheal diseases and the focus of many intervention programs (Andres et al., 2018; Waddington and Snilstveit, 2009; World Health Organization, 2014). The WASH field has often focused on household or compound-level interventions to reduce diarrheal disease risks in LMICs, but results of such interventions have been disappointing with respect to their ability to reduce disease transmission and improve health (Humphrey et al., 2019). The most promising interventions involve higher levels of community coverage for sanitation, and household connections to centralized water supply (Wolf et al., 2018). Yet despite decades of research showing that diarrhea is caused by many interdependent transmission pathways and is affected by ecological and sociological processes and the built environment, these findings have not produced substantial new research initiatives or policy decisions (Eisenberg et al., 2012b). Thus, the field could benefit from considering the larger context of pathogen transmission across communities, landscapes, and countries.

In particular, little attention has been paid to broad circulation patterns of enteric pathogens. Antibiotic resistance in enteric bacterial pathogens is an exception, where genes and/or plasmids have been used to trace movement across country or continental scales (Bokhary et al., 2021; I. Frost et al., 2019; Memish et al., 2003). Research exploring the influence of human mobility on pathogen transmission can guide WASH intervention approaches and vaccination campaigns. Information on pathogen movement within and across regions can identify new strains that may seed community outbreaks, identifying priority sites to target for control of transmission.

Combining epidemiology with genomics and phylodynamics provides a powerful approach to uncover the ways in which pathogens flow between and within communities and to examine pathogen history at relevant spatiotemporal scales (Volz et al., 2009, 2013). By leveraging genomic sequences and discrete traits (e.g., location), phylodynamics can test whether disease incidence rates match pathogen biogeography and evolutionary history (Baele et al., 2018). Increasingly, phylodynamic approaches have been used to inform public health (Rife et al., 2017). For example, in the SARS-CoV-2 pandemic, sequenced genomes are used to inform transmission dynamics (Bedford et al., 2020; Deng et al., 2020; Hamed et al., 2021), identify shifts in variant prevalence (Annavajhala et al., 2021; Hodcroft et al., 2021; Tegally et al., 2021), and determine pathogen evolutionary origins (Andersen et al., 2020; Fauver et al., 2020; Gonzalez-Reiche et al., 2020). Phylodynamic approaches have mainly been applied to RNA viruses (Ingle et al., 2021; Rife et al., 2017), in part because bacterial pathogens pose challenges due to horizontally transferred elements (e.g. genes or genomic islands) that affect evolutionary history interpretation (Brockhurst et al., 2019; McInerney et al., 2017; Vernikos et al., 2015). However, recent methods advancements in ease of whole genome sequencing and computational resources needed for phylodynamics of larger, bacterial genomes have made this more feasible(Ingle et al., 2021). Phylogeography has been used to source-track food and waterborne enteric disease outbreaks, such as *E. coli* O104: H4 or O157:H7 in produce (Buchholz et al., 2011; Cooley et al., 2007; Grad et al., 2012; King et al., 2012; Rasko et al., 2011) and *Salmonella enterica* subtypes in meat and eggs across the United States and Europe (Lienau et al., 2011; Pijnacker et al., 2019), as well as *Vibrio cholerae* in Haiti following the 2010 earthquake (Eppinger et al., 2014; Frerichs et al., 2012). Yet pathogen evolutionary history data could be used to inform an even broader perspective of disease control.

In this study, we apply phylogenomic tools to examine the potential for diarrheagenic *E. coli* seeding events in northern Ecuador. Using whole genome sequences of diarrheagenic *E. coli* isolates, we explored three main questions: 1) Do we observe signatures of either urban or rural ancestry across diarrheagenic *E. coli*?; 2) Are *E. coli* pathotypes geographically structured?; and 3) Are there directionally elevated rates of migration between urban to rural areas, or vice versa? Our results of biogeographic patterns of diarrheagenic *E. coli* provides insights that can inform national public health strategies for bacterial enteric pathogens.

## 2. Materials and methods

### 2.1. Sampling and design

**Fig. 1.**
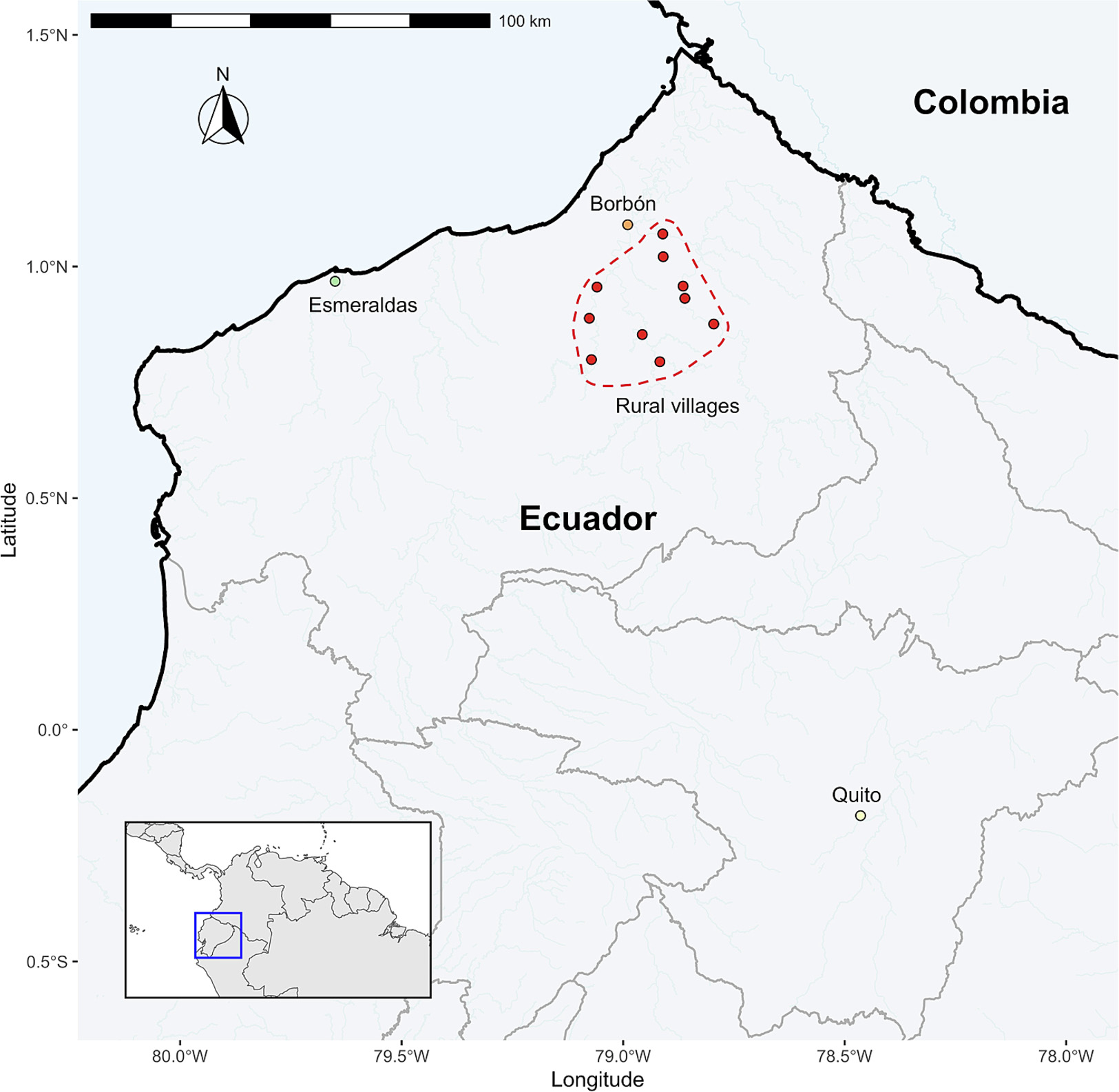
Map of study sites. Location of the study sites across an urban-rural gradient in northern Ecuador: Quito (population ~ 1.6 million), Esmeraldas (population ~ 162,000), Borbon (population ~ 5000), and rural villages (outlined by dashed lines) (population ~ 50–500 per village). Inset map highlights the country of Ecuador within Central and South America.

Samples were collected during EcoZUR (*E. coli en Zonas Urbanas y Rurales*), an age-matched case-control study of diarrhea carried out at four sites across an urban-rural gradient in northern Ecuador. Detailed methods for this study can be found in Smith et al. (2019). The four main study locations were: Quito (the nation’s capital) with a population of roughly 1.6 million people; Esmeraldas, a coastal city with a population of 162,000; Borbón, a town within the Esmeraldas providence with an estimated population of ~7000 people; and rural villages distributed along three main rivers: the Santiago, the Cayapas, and the Onzole, with anywhere from 50 to 500 people in each village. The study was designed to investigate how factors such as population density, WASH conditions, animal contact, and human travel affect the incidence of diarrheagenic *E. coli* across this urban-rural gradient. Quito and Esmeraldas were designated ‘urban’ sites while Borbón and the remaining rural communities were designated as ‘rural’ sites, as in previous analyses (Soto-Girón et al., 2021) (Fig. 1). ‘Urban’ locations are characterized by greater population density, water and sanitation infrastructure, and access to roads and medical facilities compared to ‘rural’ locations.

### 2.2. Participant recruitment

Study participants were recruited between April 2014–September 2015 from either Ecuadorian Ministry of Health (MSP) hospitals or local clinics at each of the sites (Centro de Salud N°4 Chimbacalle in Quito, Hospital Delfina Torres de Concha in Esmeraldas, and Hospital Básico de Borbon in Borbón). Rural village participants were recruited through MSP clinical visits or at Borbón Hospital when they visited for medical attention. Cases comprised individuals who self-reported diarrhea, defined as three or more loose stools in a 24-h period without antibiotic intake over the previous seven days from sampling day. Controls were recruited from the same facility and defined as individuals presenting with a non-diarrheal illness, without diarrhea or vomiting in the prior seven days. A one-to-one age-match of cases and controls was carried out in real-time, with age-matching. Both case and control participants were required to be a resident of the study location for at least six months and were excluded if they reported antibiotic usage in the prior week(S. M. Smith et al., 2019).

After obtaining informed consent, participants completed an electronic survey including patient/household demographics, socioeconomic status, medical history, WASH practices, and travel history (over the past week, month, and year). Participants were asked for a stool sample, which was streaked for isolation of *E. coli* in the field laboratory and re-isolated for pure cultures at Universidad San Francisco de Quito (USFQ), except in Quito, where Cary-Blair transport media swabs (BD, Franklin Lakes, NJ) were inoculated with fecal material and streaked for isolation at USFQ; swabs were maintained at 4 °C for a maximum of 48 h. Of the 907 participants successfully enrolled with a completed the survey, 85% (*n* = 771) provided a stool sample.

### 2.3. Domestic travel

Self-reported travel was defined as any mobility event where the participant left their origin city/village with a different destination within Ecuador. Common destinations in surveys included Guayaquil, Quito, Santo Domingo, Esmeraldas, San Lorenzo, Borbón, and rural villages. Participants also had the option of inputting other destinations. The survey included temporal intervals of travel within the last week, month, and year. We previously reported that rural participants (Borbón, 67%; rural villages, 63%) had higher rates of travel than urban participants (Quito, 22%; Esmeraldas, 33%), and travelers to urban regions had higher risk of diarrhea and pathogenic *E. coli* infections (S. M. Smith et al., 2019).

### 2.4. Ethics

Use of human subjects and permission for this study was approved through the Ecuadorian Ministry of Public Health (MSP-DIS-2014–0055-O), Emory Institutional Review Board (IRB) (IRB00065781) and the USFQ Ethical Committee (2013–145 M).

### 2.5. E. coli isolation and PCR screening

For each fecal sample, five lactose-fermenting isolates were streaked and cultured on MacConkey Lactose agar and non-lactose-fermenting isolates were cultured on Chromocult agar media (Merck, Darmsladt, Germany) to test for β-glucoronidase activity. Non-lactose fermenting isolates were biochemically tested using an API 20E assay (BioMérieux, Marcy l’Etoile, France) for identification as *Shigella* or *E. coli*. The five lactose-positive isolates were pooled and resuspended in 300 μL of sterile water, boiled for 10 min to release DNA, and the resulting supernatant was used for PCR testing. Using nine different primer sets, pooled isolates were used for singleplex colony PCR detection of virulence genes characteristic of diarrheagenic *E. coli* pathotypes (for details see Smith et al. (S. M. Smith et al., 2019)). Isolates from positives pools were tested individually for the gene(s) that tested positive. A total of 316 *E. coli* isolates tested positive for at least one of the nine PCR assays.

### 2.6. DNA sequencing

DNA from *E. coli* colonies that were positive for a pathotype-specific gene were extracted using Wizard Genomic DNA purification kits (Promega, Madison, WI). DNA concentrations were estimated using both NanoDrop spectrophotometer (Thermo Scientific) and Qubit 2.0 double stranded DNA high-sensitivity assay (Invitrogen, Carlsbad, CA). Sequencing libraries were made using Illumina Nextera XT DNA library preparation kits according to manufacturer recommendations. Library insert sizes were determined through a Bioanalyzer 2100 (Agilent) on a High Sensitivity DNA chip. Equimolar concentrations of libraries were sequenced on an Illumina MiSeq (MiSeq reagent v2 kit for 500 cycles, 2 × 250 bp paired end) (Illumina, Inc.). Adapters were trimmed and demultiplexed by sample using MiSeq control software v2.4.0.4. A total of 279 isolates were successfully sequenced. Raw reads were deposited in NCBI Sequence Read Archive (BioProject ID PRJNA486009).

### 2.7. Read quality control and genome assembly

Post sequencing, reads were screened, trimmed, and cleaned using software tools in the Microbial Genomes Atlas (MiGA) pipeline (Rodriguez et al., 2018). Specifically, adapter sequences were removed from raw reads using Scythe v. 0.994 (https://github.com/vsbuffalo/scytheReads) and reads were trimmed at both 5′ and 3′ ends based on PHRED score cutoff of 20 using SolexaQA++ v. 3.1.7.1 (Cox et al., 2010). Any reads ≤50 bp post trimming were discarded. Cleaned and trimmed reads were de novo assembled using MiGA-implemented Iterative de Bruijn Graph Assembler for sequencing data with highly uneven depth (IDBA-UD) v. 1.1.3 (Peng et al., 2012).

### 2.8. E. coli pathotype whole-genome confirmation

A read-based gene content analysis of whole-genome sequencing data for each isolate was used to confirm the PCR-based *E. coli* pathotype designations. This approach was chosen over traditional homology-based designations to avoid the limitations of the assembly process, such as gaps, truncated genes, or mis-assemblies. Both reference genes and pathotype inclusion criteria are summarized in S1 Table.

To conduct the read-based gene content analysis, we mapped high-quality, trimmed reads against a reference pathotype gene sequences using nucleotide BLAST (blastn) (Camacho et al., 2009). We then filtered the blastn output to include only reads with ≥95% query sequence identity and ≥ 80% query length coverage. Next, the script “BlastTab. seqdepth_ZIP.pl” from the Enveomics collection (http://enve-omics.ce.gatech.edu/enveomics/) was used to calculate the observed sequencing depth as well as the number of reads mapping to each gene in the pathotype database. Gene presence/absence was determined by the number of reads recruited (i.e. depth of coverage) and the percentage of the gene length that was covered (i.e. breadth of coverage), assuming a zero-inflated Poisson distribution to correct for non-covered positions. Genes with zero inflation values of ≥0.3, which represents the fraction of the gene that is not covered, were excluded. Thus, only genes with at least 70% breadth of coverage were considered to be present. Isolates were assigned a given pathotype designation if the PCR-based and whole-genome pathotype results matched. If there was no genome-based confirmation of the PCR-based pathotype, we labelled the isolates as “Undetermined”. Finally, only isolates with corresponding participant travel survey data (*n* = 260) were included in the following analyses.

### 2.9. Phylogenomics

To explore relationships between isolates, we inferred two phylogenomic trees to be used in subsequent analyses. First, a pan-genome tree was created using Roary v. 3.13.0 (Page et al., 2015), using all orthologous gene sequences to create a concatenated alignment across all genome assemblies (hereinafter “core genome tree”). We recovered a total of 1030 genes (present in 99%–100% of isolates) for the core genome tree out of 82,704 genes found by Roary. Despite this being only a small percentage of the *E. coli* genome (1.2%) this represents a conservative and orthologous set of genes to adhere to phylodynamic model assumptions. To prepare assemblies for Roary, we annotated our assembled contigs using Prokka v. 1.14.5 (Seemann, 2014) which outputted each genome in general feature format (GFF3). We then ran Roary with default parameters. Using the core gene alignment fasta output from Roary, we removed spurious sequences and regions of poor alignment with trimAL v. 1.4.rev22 (Capella-Gutiérrez et al., 2009) then inferred a core gene phylogenetic tree with IQ-Tree v. 2.1.4 (Nguyen et al., 2015) implementing ModelFinder (Kalyaanamoorthy et al., 2017) and 1000 ultra bootstraps (Hoang et al., 2018). We used ModelFinder’s suggested Generalized Time Reversible (GTR) model with empirical base frequencies, allowing for a proportion of invariable sites, and discrete Gamma model with default 4 rate categories (GTR + F + I + G4) to infer the final core genome tree.

Using the inferred core genome tree, we tested for phylogenomic signal across the urban and rural gradient. We evaluated the significance of a phylogenomic signal using Borges’ δ-statistic(Borges et al., 2019), which measures the degree of phylogenomic signal between a categorical trait and a phylogeny. We calculated the observed δ-value in our data using default parameters (rate parameter = 0.1, standard deviation = 0.5, number of iterations = 10,000, thinning = 10, and burn-in of iterates = 100). To assess significance, the δ value was compared to 1000 replicates of randomizing our urban/rural trait along the phylogeny. Significance was calculated as the number of random simulations where the δ value was higher than our δ real value with a resulting *p*-value.

The second phylogenomic tree incorporated a single nucleotide polymorphism (SNP)-based approach (hereinafter “SNP tree”) using Snippy v. 4.6.0 (https://github.com/tseemann/snippy). Snippy used the same input of assembled contigs as described above for the core genome tree. Using core SNPs identified from Snippy, we searched for regions under recombination using Gubbins v. 3.0.0 (Croucher et al., 2015), which identifies loci containing increased densities of base substitutions suggestive of recombinant regions. Four isolates were pruned from the original 260 based on significant character composition deviating from the average composition of the alignment based on Gubbins default parameters. We extracted polymorphic sites outside of recombination regions identified in Gubbins using snp-sites v. 2.5.1 (Page et al., 2016). We used the subsequent SNP alignment for subsequent ancestral spatial reconstruction and structured coalescent approaches. For all analyses above we used *E. coli* SE11 reference genome, which is representative of a commensal gut *E. coli*(Oshima et al., 2008).

### 2.10. Discrete spatial reconstruction

To estimate ancestral spatial locations of *E. coli* isolates, we first inferred a maximum likelihood unrooted tree with our recombinant-free SNP alignment using a GTR model in iqTREE v. 2.1.4 with 1000 ultra bootstraps (Nguyen et al., 2015). For our SNP-based approach, we included 256 isolates and recovered 1254 SNP sites within the core alignment, while controlling for regions of recombination. Ancestral spatial locations of *E. coli* isolates were then reconstructed using this maximum likelihood SNP tree. The SNP tree was annotated with an ‘urban’ or ‘rural’ designation through PastML v. 1.9.15 (https://pastml.pasteur.fr/) (Ishikawa et al.,2019). PastML is a decision-theory based method to associate nodes within the phylogeny to predict sets of likely states. Working from tips to the inner nodes of the tree, PastML predicts states with uncertainty to assess likelihood of a unique state. Using default parameters, we estimated ancestral states of either ‘urban’ or ‘rural’ across our SNP tree with a marginal posterior probability approximation (MPPA) method and the Felsenstein-81 substitution model (Felsenstein, 1981), which incorporates accurate ancestral predictions (Ishikawa et al., 2019). The resulting predictions and phylogeny were visualized and annotated using Interactive Tree of Life v. 6.3 (Letunic and Bork, 2021).

### 2.11. Migration

Using our maximum likelihood SNP tree, we assessed the level of temporal phylogenetic signal in our data by plotting root-to tip divergence against sampling date using TempEst v. 1.5.3(Rambaut et al., 2016). Due to limited temporal signal in our data (S1 Figure), we used subsequent analyses for relative relationships rather than specific dates among discrete traits, node timing, and used informed priors for rates of substitution from past studies of pathogenic *E. coli*.

We inferred the relative migration rates between our urban and rural populations using the Marginal Approximation of the Structured Coalescent (MASCOT) package in BEAST2 v. 2.6.4(Bouckaert et al., 2019) for the SNP alignment. MASCOT applies an approximate structured coalescent model to estimate the marginal probability that a randomly chosen lineage in a previous generation came from a population different from the one in which it is currently found in the preceding generation(Müller et al., 2018). A lineage’s state (e.g., either urban or rural) is calculated backwards on the phylogeny with all potential migration events but explicitly based on the probability of coalescence (i.e., a lineage will not migrate if it never coalesces). By using an approximate structured coalescent model, we can explore the relative, pairwise rates of migration between localities. Given that we did not sample all possible locations in the country, we also applied a third ghost population, categorized as “outside”, to infer whether isolates may have greater migration from unsampled locations. There were no samples used to create the outside “ghost” population and this population was used only as an additional source.

Given BEAST2 expects a full genome alignment and we used a concatenated alignment of SNPs outside of recombinant regions, we corrected our BEAST2 file to include the number of constant nucleotide sites across the *E. coli* genome. Using the total number of nucleotide sites within an *E. coli* reference genome (https://www.ncbi.nlm.nih.gov/nuccore/556503834) compared to our filtered variant call format (VCF) we used beast2_constsites (https://github.com/andersgs/beast2_constsites) to calculate and add the number of constant nucleotide sites to our BEAST2 file. We built our MASCOT model with a GTR plus discrete gamma with four states (GTR + _Γ_4) substitution model, a strict clock model, substitution rate prior of 6.90 × 10^−7^ mutations per base per year based on observed gut *E. coli* rates(Ghalayini et al., 2018), and Constant Bayesian Search Variable Selection for the MASCOT tree. Three independent analyses were performed with 100 million Markov Chain Monte Carlo (MCMC) steps, recording samples every 5000 MCMC steps, with a 10% burn-in period. We combined the three independent runs and verified that the collective effective sample size for all migration and effective population size parameters was at least 150. We also reran the above parameters to test our threshold for recovery of migration rates and potential migration rate changes for: 1) fixed effective population size, 2) clinical diarrhea status by selecting a random subsample (*n* = 50) of either “case” or “control” clinical status with fixed effective population size, and 3) without including a “ghost” population. Except for mentioned changes, all runs used identical BEAST2 parameters. Post MASCOT model runs, we used TreeAnnotator v. 2.6.3 and LogCombiner v.2.6.6(Bouckaert et al., 2019) to summarize migration rates among populations.

## 3. Results

With the final set of *n* = 260 diarrheagenic *E. coli* isolates that were sequenced and had corresponding participant travel survey data available, we recovered a total of 1030 genes (present in 99%–100% of isolates) for the core genome tree. For our SNP-based approach, we included 256 isolates and recovered 1254 SNP sites within the core alignment, while controlling for regions of recombination. We present cumulative results of both phylogenies, including signals of biogeographic structuring and migration rates among isolates.

### 3.1. Extensive mixing of E. coli genotypes

**Fig. 2.**
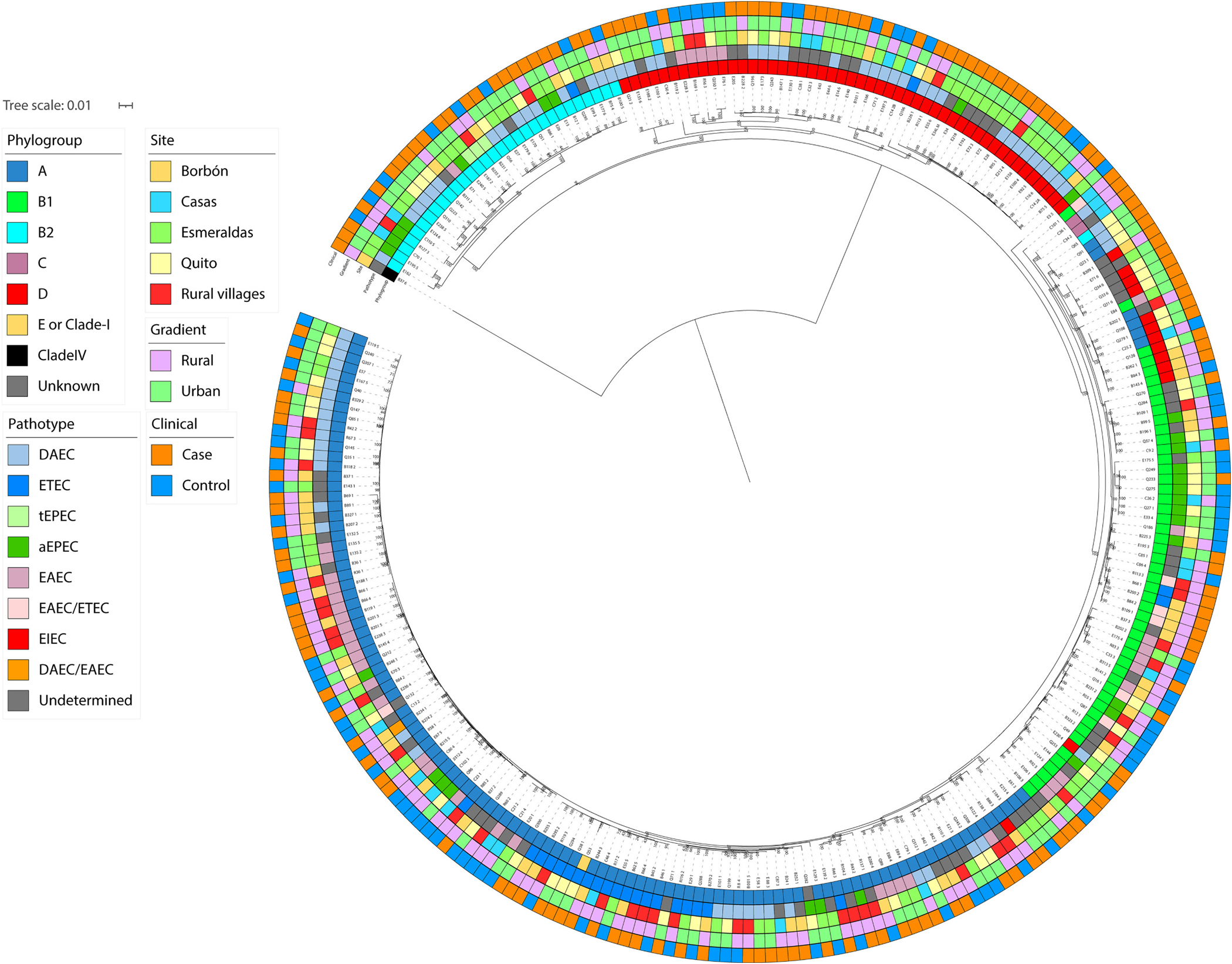
Roary unrooted core genome phylogeny. Tips represent 260 whole genome isolates of diarrheagenic *Escherichia coli* (*E. coli*) across clinical status (case vs control), pathotype (determined via PCR-based and genomic analysis), specific site, and urban-rural location. Pathotypes include diffusely adherent *E. coli* (DAEC), enterotoxigenic *E. coli* (ETEC), typical enteropathogenic *E. coli* (tEPEC), atypical enteropathogenic *E. coli* (aEPEC), enteroaggregative *E. coli* (EAEC), and enteroinvasive *E. coli* (EIEC). Isolate pathotypes are ‘Undetermined’ if there was no genome-based confirmation of the PCR-based pathotype.

Using the core genome tree, we observed little-to-no signal of *E. coli* isolates structuring by individual site, urban rural locality, or defined pathotype (Fig. 2). When subclades clustered by specific metadata, such as a clinical status, pathotype, or site, they were typically discordant from other metadata variables. For example, isolates clustered by site within certain subclades but were represented by multiple pathotypes (and vice versa). Using the Borges’ δ value, we found a trending but non-significant phylogenomic signal across our urban-rural trait (*p* = 0.07, δ-real = 0.57, δ-simulated = 0.24 ± 0.28 SD).

**Fig. 3.**
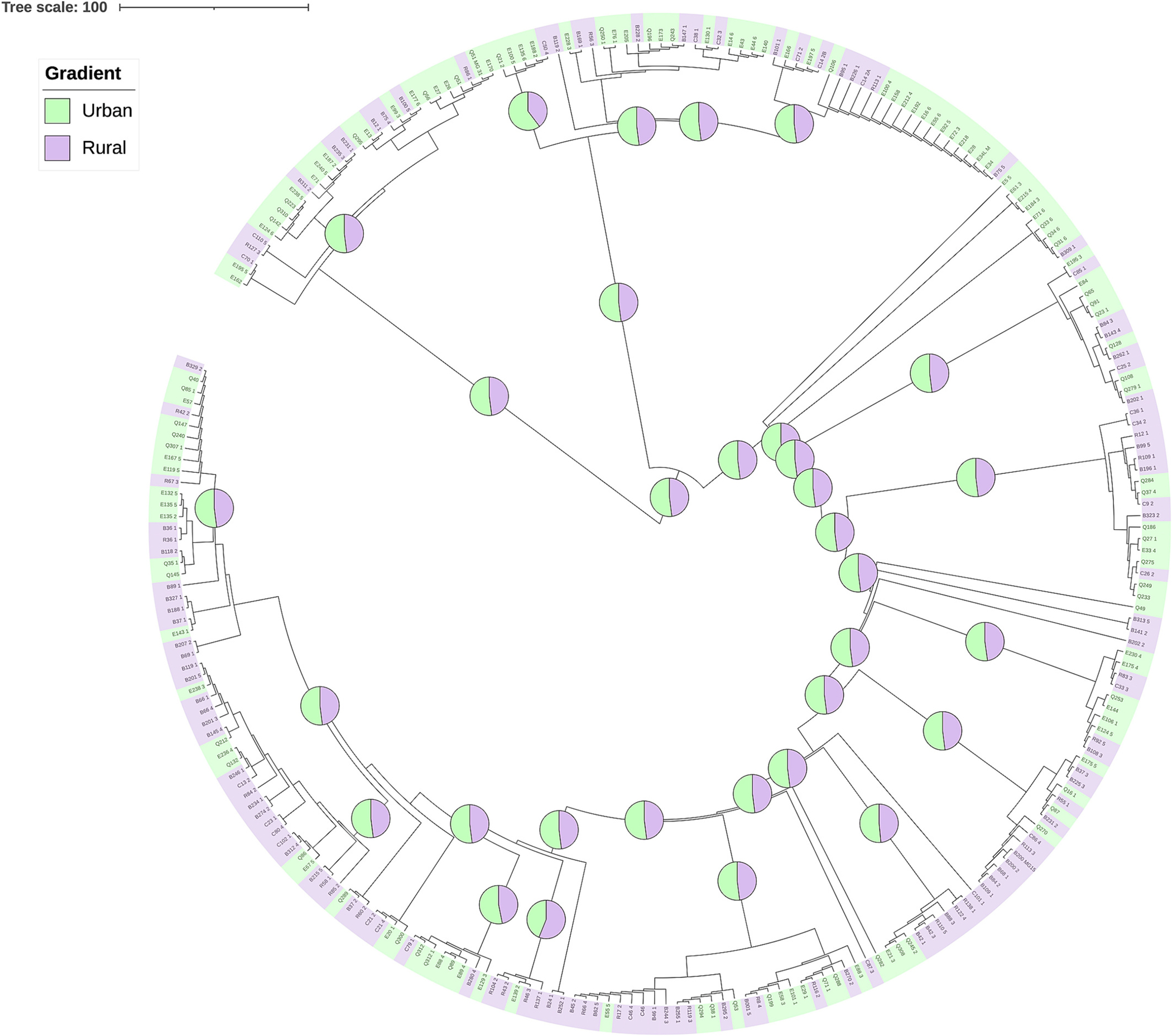
PastML-based discrete spatial reconstruction of urban and rural localities across a maximum likelihood SNP phylogeny. Tips colored by sampled location of urban or rural. Pie charts on ancestral branches represent percent confidence (out of 100%) of the marginal posterior probability approximation (MPPA) method and the Felsenstein-81 substitution model of either rural or urban ancestry.

Mixing of *E. coli* genotypes was also supported by the discrete ancestral reconstruction (Fig. 3). Assigning isolates to ‘urban’ or ‘rural’ suggested mixed ancestral states for nodes closest to the base of the SNP tree. Mean probabilities across all nodes and tips were 51% for urban ancestry and 49% for rural ancestry (S2 Table). While some nodes supported either urban or rural states being dominant, ancestral assignments frequently switched to opposing directions before reaching the tips of the phylogeny. Given the lack of support for either an urban or rural ancestral state, the SNP tree suggests patterns of extensive mixing of *E. coli* genotypes across the study area.

### 3.2. Equivalent measures of migration rates across urban-rural gradient

**Fig. 4.**
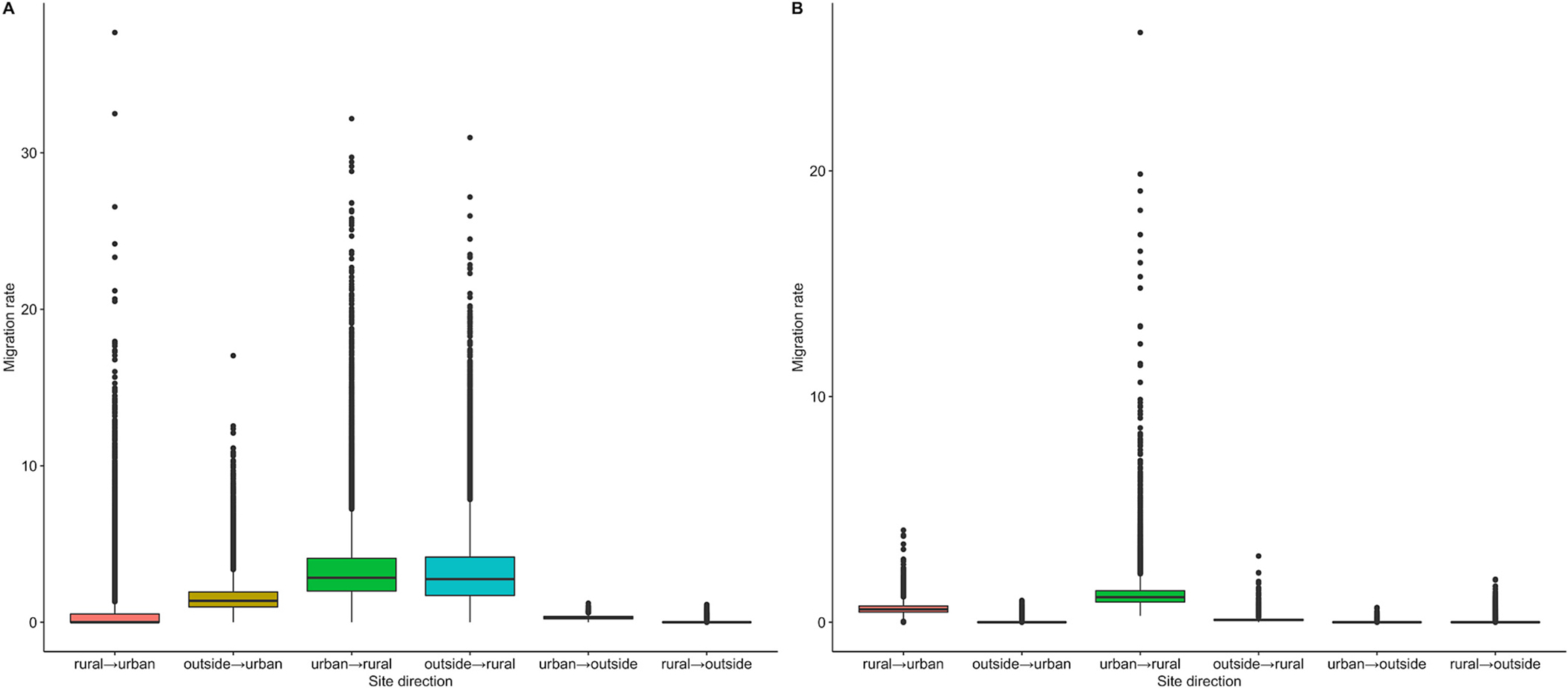
MASCOT migration rates (as a coefficient of transmission events per year) by pairwise population population directions (rural, urban, and “outside”). A. Allowing for inference of effective population size. B. Controlling for effective population size. Boxplots represent variation of all sampled migrations rates for entirety of MASCOT BEAST2 run. Each box plot displays mean (horizontal line), first and third quartiles (bottom and top of box), minimum and maximum values within inter-quartile range of the lower and upper hinges (vertical lines) and outliers (points).

To further explore patterns of *E. coli* biogeography, we estimated the direction and rate of migration between urban and rural populations. Using an approximate Bayesian structured coalescent approach, we estimated that *E. coli* isolates had migration rates highest from urban towards rural populations and equivalent migrations rates from unsampled “outside” populations towards rural populations (Fig. 4). Rates of migration (measured as number of transmission events per year) were estimated to be 6.7-times larger for urban to rural (mean migration rate coefficient 3.3 ± 2.1 SD) over rural to urban (mean migration rate coefficient 0.5 ± 1.2 SD). Rates of migration from outside to rural were also 7.2-times larger (mean migration rate coefficient 3.3 ± 2.2 SD) over rural to urban (Fig. 4a). When controlling for effective population size (setting a constant for each population) we observed a similar differential of migration rates with urban to rural being higher than rural to urban locations (Fig. 4b). Lastly, we found similar migration rate trends within randomly selected diarrheal cases versus asymptomatic controls (S2 Figure) as well as when removing “outside” populations (S3 Figure). However, when removing for “outside” populations, BEAST2 models were unable to converge under similar parameters.

### 3.3. Mixing of E. coli pathotypes within clades

**Fig. 5.**
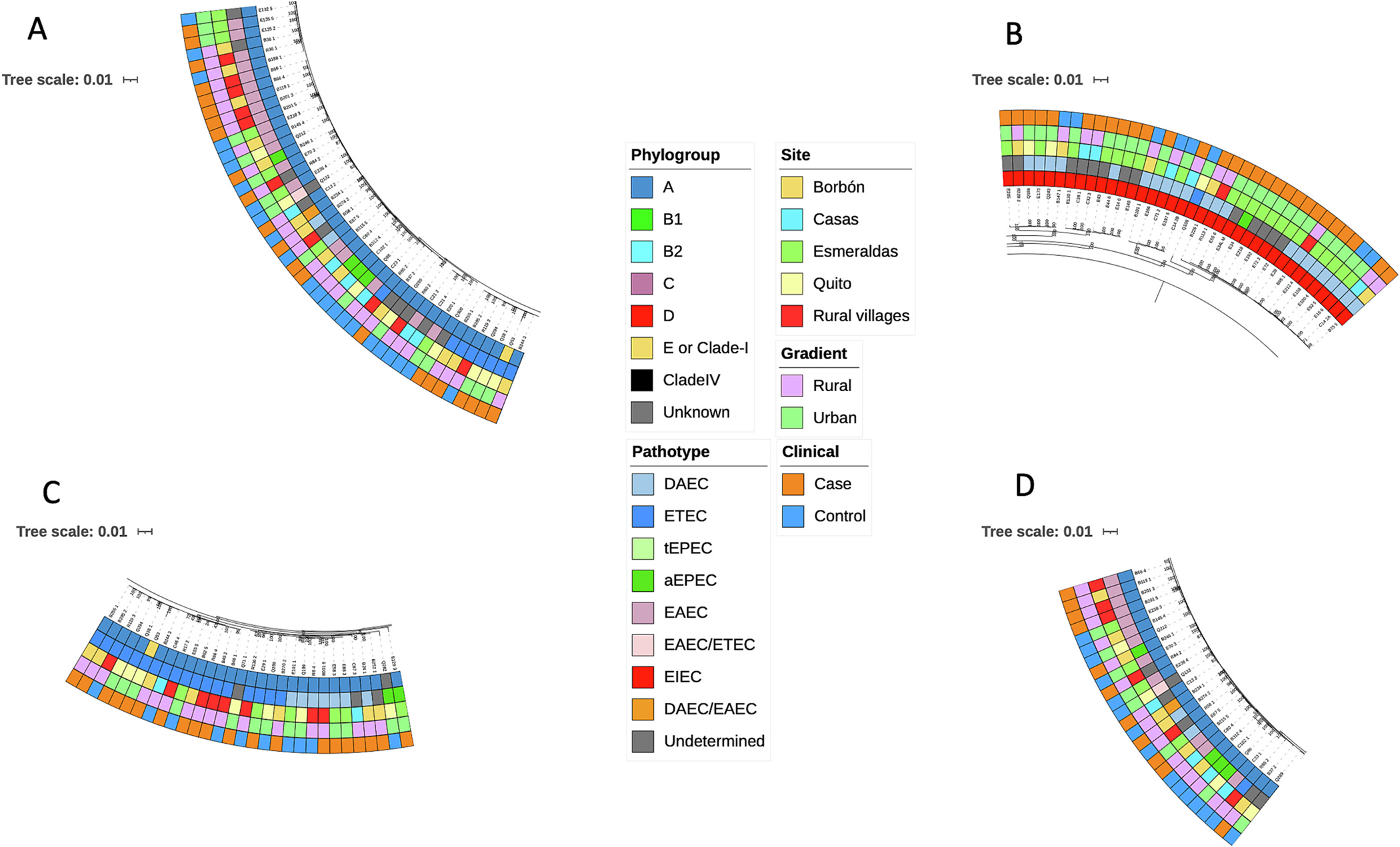
Clades of mixed pathotypes and/or site locations. A) Subclades representative of strains mixed by both location and pathotype B) Subclades representative of strains with the same pathotype isolated from multiple locations C) Additional example of subclades representative of strains with the same pathotype isolated from multiple locations D) Subclade with different pathotypes isolated from the same location: example of paired samples (B234_1 and B274_2 indicated with star).

Given the lack of spatial structuring or definitive ancestral state of either urban or rural across our phylogenies, we explored, qualitatively, how isolates may be evolutionarily related but different across key metadata variables. Across our core genome tree (Fig. 2), we found specific examples where diarrheagenic *E. coli* isolates have interesting patterns belying extensive mixing: multiple pathotypes (e.g. EAEC, ETEC, and EPEC) were located on the same clade but these isolates came from different spatial locations (e.g. urban Quito and rural Borbón) (Fig. 5a; subclades with strains mixed by both location and pathotype). We also observed isolates from different spatial locations that had core genomes that were evolutionarily similar, such as DAEC in Fig. 5b and ETEC in Fig. 5c (subclades with the same pathotype isolated from multiple locations). Lastly, there were examples of phylogenomic clusters that were isolated from similar sites, such as two paired samples from Borbón (B234_1 and B274_2) that are characterized by different pathotypes (EIEC and DEAC, respectively) (Fig. 5d; subclades with different pathotypes isolated from the same location).

## 4. Discussion

This study provides a phylogenomic framework to describe the distribution and movement of diarrheagenic *E. coli* across northern Ecuador. Our main result, that *E. coli* isolates lack clustering based on location, pathotypes, or along our urban-rural gradient, indicates that there is substantial transmission of bacterial enteric pathogens in northern Ecuador, and that new strains are regularly introduced between sites across the country. While we observed high pathogen connectivity between all communities sampled, we also observed that rates of migration for *E. coli* strains were largest in the direction of urban towards rural populations. Previous analysis of EcoZUR study data has demonstrated that more rural travelers visit urban spaces (S. M. Smith et al., 2019), and the results presented here support the hypothesis that urban localities may be seeding rural communities with novel diarrheagenic *E. coli* strains.

Diarrheal disease research has traditionally focused on individual, household, and community scales to understand localized risk factors for disease transmission (Eisenberg et al., 2012b), but larger scale factors such as climate and human travel are also critical in determining the spread of enteric diseases. Here, by analyzing fine scale genomic sequence data, we can broaden the scale of analysis at which enteric disease epidemiology traditionally takes place, expanding considerations of localized factors to an examination of country-wide drivers of enteric disease transmission. Human movement can increase disease transmission via infectious individuals entering a naïve population or via susceptible hosts traveling to an endemic region. This social connectivity can be considered as a feature of the “landscape,” similar to the flow of water or air in disseminating pathogens (Biek and Real, 2010).

Analyses combining social, epidemiological, and molecular data to gain insights about infectious disease transmission processes on a country-wide scales have important public health consequences. Studies on spatial disease dynamics of measles in Niger showed that, while localized extinctions in measles occur, agricultural seasonality, contact clusters, and road networks that facilitate host movement can result in recurrent seasonal epidemics and will sustain transmission if left unaddressed (Bharti et al., 2010, 2012). Social-network analysis with high resolution genome sequencing enhanced the investigation of a tuberculosis outbreak by identifying key nodes in the transmission network to target for interventions(Gardy et al., 2011). Molecular, genetic, and social network analyses have been used to differentiate chlamydia genotype clusters on the basis of sociodemographic information (Wylie et al., 2005). In this study, the result that urban-derived strains may be finding their way to rural areas could point to a country-wide strategy focused on improving WASH conditions and/or strengthening vaccination campaigns in urban areas.

### 4.1. Lack of spatial structure indicates high pathogen connectivity between communities

Our observed extensive mixing of *E. coli* isolates is indicative of a high transmission environment. Ecuador, classified as high-middle socio-demographic index (SDI) country, has substantially lower rates of diarrhea than many other countries (diarrhea represents 2.72% of total disability-adjusted life years (DALYs) for children <5 in Ecuador, compared to 12.28% across low SDI countries) (Institute for Health Metrics and Evaluation, 2019). Even so, the short branch lengths in our core genome tree (Fig. 2) are suggestive of recent or ongoing strain mixing, and not specific strains emerging and becoming dominant, as in genetically diverse pathogens like HIV (Hemelaar, 2012) or other bacterial pathogens (Achtman, 2016). Especially in instances of *E. coli* outbreaks in high-income settings, inferred phylogenies are typically well-structured by specific metadata (host or location) (Cheung et al., 2011; Dallman et al., 2021; González-Escalona and Kase, 2019), and therefore can be traced to origin location, source, or host (Buchholz et al., 2011; Cooley et al., 2007; Gieraltowski et al., 2016; Gilpin et al., 2020; Hoffmann et al., 2016; King et al., 2012; Lienau et al., 2011; Nenonen et al., 2012; Pijnacker et al., 2019; Prystajecky et al., 2015; Rasko et al., 2011; Tsui et al., 2018), possibly due to lower overall transmission rates of diarrheagenic *E. coli* in these populations. We do not observe high levels of genetic structuring and, generally, pathogens with differing levels of population structure (high vs. low) suggest certain factors (e.g. host or location) are promoting or impeding rates of gene flow, which can directly correspond to transmission rates (Andam et al., 2017; Pybus et al., 2015). Thus, our work may also be generalizable to regions with similar or even greater sustained levels of enteric pathogen transmission, where extensive mixing may also occur, depending on the mobility of the human population.

### 4.2. Rates of pathogen migration suggest urban towards rural movements

Elevated travel rates among people from rural communities to urban areas are a plausible explanation for observed higher migration rates from urban towards rural sites. People traveling to urban areas for work, health care, or leisure are entering higher diarrheal transmission areas and, once infected, may bring pathogenic strains back to rural communities (S. M. Smith et al., 2019). Additionally, the “outside” population (categorized as unsampled localities in our study) also suggests migration towards rural communities. New strains of *E. coli* could enter the community, region, or country through urban routes (highways, airports, train stations, etc.) and may explain unsampled locations as other sources for diarrheagenic *E. coli* strains. Studies of pertussis in Senegal (Broutin et al., 2004) and meningococcal disease in Niger (Bharti et al., 2012) have found more densely populated areas are known to seed outbreaks to rural communities.

Our previous work in the region also supports this hypothesis. In the EcoZUR study we identified increased risk of diarrheal diseases of participants traveling from rural communities to urban city centers (S. M. Smith et al., 2019). We also found road building was associated with increased risk of diarrheal disease, particularly bacterial enteric pathogens, across these communities, with higher risk in the more urban site (Bates et al., 2007; J. N. S. Eisenberg et al., 2006). Patterns of antibiotic resistant genes in the region due to human behaviors suggest an increased opportunity for *E. coli* strains to acquire pathogenic traits across more urban environments and communities (Eisenberg et al., 2012a). A study focused on the prevalence of enteroinvasive *E. coli* (EIEC) and enterotoxigenic *E. coli* (ETEC) in this region found that persistence of EIEC and ETEC may be related to connectivity to both Colombia and other regions of Ecuador (Bhavnani et al., 2016). Future work could benefit from additional field-based longitudinal sampling of *E. coli* to directly test migration rates.

### 4.3. Public health implications

We demonstrate here that a regional-scale phylogenomic framework for enteric pathogen transmission can be used to identify focus areas for larger-scale WASH intervention strategies. Improving WASH conditions has historically been tied to safe water supply and sanitation systems, yet there has been a shift from the public to the private domain in the WASH sector(Eisenberg et al., 2012b). Increased emphasis has been placed at the household, through interventions such as hygiene education and household water treatment, and recent WASH intervention trials focused on rural settings(Humphrey et al., 2019; Luby et al., 2018; Null et al., 2018; Pickering et al., 2019b) where randomized control trials are more easily implemented(Levy and Eisenberg, 2019; Pickering et al., 2019a). However, evaluations of these trials have failed to identify an effect, and the impact of household-scale interventions has been called into question, due to issues related to uptake, acceptability, compliance, scalability, durability, and effectiveness (B. Arnold et al., 2009; B. F. Arnold and Colford, 2007; Luby et al., 2008; Mäusezahl et al., 2009; Rosa and Clasen, 2010; Schmidt and Cairncross, 2009). It is becoming increasingly doubtful that household-level interventions can take the place of public infrastructure projects targeting larger populations.

Our results suggest that controlling pathogens in dense population centers could also affect conditions in outlying rural areas by limiting regional circulation of diverse strains of diarrheagenic *E. coli*. Reducing enteric transmission in urban areas through WASH infrastructure and/or vaccination campaigns represents a more efficient way to reach larger concentrated populations, that may have downstream effects on rural populations, rather than focusing intervention efforts on diffuse rural populations. This strategy may be especially important given trends towards increasing urbanization in LMICs (Alirol et al., 2011).

### 4.4. Limitations

Limitations of our study highlight opportunities for future research. This study was limited to four sites, and we were unable to sample from other locations in Ecuador (notably the large urban population of Guayaquil). For this reason, we included the outside population in the migration analysis to capture the contributions of unsampled sites. Another limitation was our inability to find temporal signals in our dataset, an indication that bacterial genomic datasets collected within a few months are likely inadequate for specific node timing inference (Rambaut et al., 2016). Our case-control experimental design carried out at multiple sites during the same time period allowed us to compare risk factors for both symptomatic and asymptomatic diarrhea but was also cross-sectional. Future research efforts in this area should use a longitudinal study design that more closely matches the rates of evolution for bacterial pathogens, on the order of years rather than months. Being able to recover temporal signals for fine-scale investigations of disease transmission would allow for analysis of the specific timing and tempo of bacterial pathogen spread in the region and better inform intervention strategies.

Sampling bias is also a common concern in phylodynamics models and can impact results if ignored (De Maio et al., 2015; S. D. Frost et al., 2015; Kühnert et al., 2011; Moncla et al., 2021). In our study, we explicitly used structured coalescent phylogenetic frameworks, which are relatively robust to sampling bias. Forcing sampling by equivalent effective population sizes and not inferring specific transmission events showed promising results for effectively implementing phylodynamic approaches (Müller et al., 2018; Vaughan et al., 2014). Additionally, by recapitulating migration rate trends with a random subset of our samples (*n* = 50 for both symptomatic and asymptomatic participants), we show that these models are robust to variable sample sizes.

An additional limitation was reconciling the evolutionary complexities of bacterial species pangenomes. For rates of evolution models, ignoring or removing regions under significant recombination is critical to avoid breaking model assumptions (Croucher et al., 2015; Didelot and Maiden, 2010). In cases of bacterial pathogens, parts of the pangenome that are likely most important for defining strain characteristics (pathotype, virulence, etc.) are located in dynamic regions of the accessory genome (Brockhurst et al., 2019; McInerney et al., 2017). Building phylodynamic models both applicable to broader pathogen types (e.g., bacterial, fungal) and other modes of evolutionary change (e.g., pangenomes, horizontal gene transfer, recombination) would represent a significant advance for the field, that could increase our understanding of pathogen dynamics, especially for pathogens that have larger genomes and different transmission modes. This is beyond the scope of the current study but would have immediate implications for pathogens of public health concern.

## 5. Conclusions

Combining traditional epidemiological variables of social, economic, demographic, and behavioral factors with genomic data has revolutionized how, where, and when to implement strategies to mitigate disease outbreaks. We show that leveraging enteric bacterial pathogen phylogeography and evolutionary history to inform public health priorities can and should be applied beyond outbreak response in LMIC settings with high burden of diarrheal disease. By incorporating these approaches, we can shed light on broad transmission patterns and tease out important disease dynamics at varying spatial and temporal scales. Finding broader use cases for phylodynamic approaches in public health will help expand regional disease contexts and complement traditional, localized efforts at the individual, household, and community scales.

## Supplementary Material

2

3

1

4

6

5

## Data Availability

Raw isolate reads can be found in NCBI Sequence Read Archive BioProject ID PRJNA486009. Roary outputs and phylogeny, SNP alignments and phylogeny, BEAST2 xml and output log files, PastML output, processed data, and isolate metadata available at https://doi.org/10.6084/m9.figshare.19232412.v1.
