# Supplementary figures and images for: Population genomics of diarrheagenic Escherichia coli uncovers high connectivity between urban and rural communities in Ecuador
Source: Infect Genet Evol. Author manuscript; Available in PMC 2023 Oct 25. (PMC10599324; doi:10.1016/j.meegid.2023.105476)

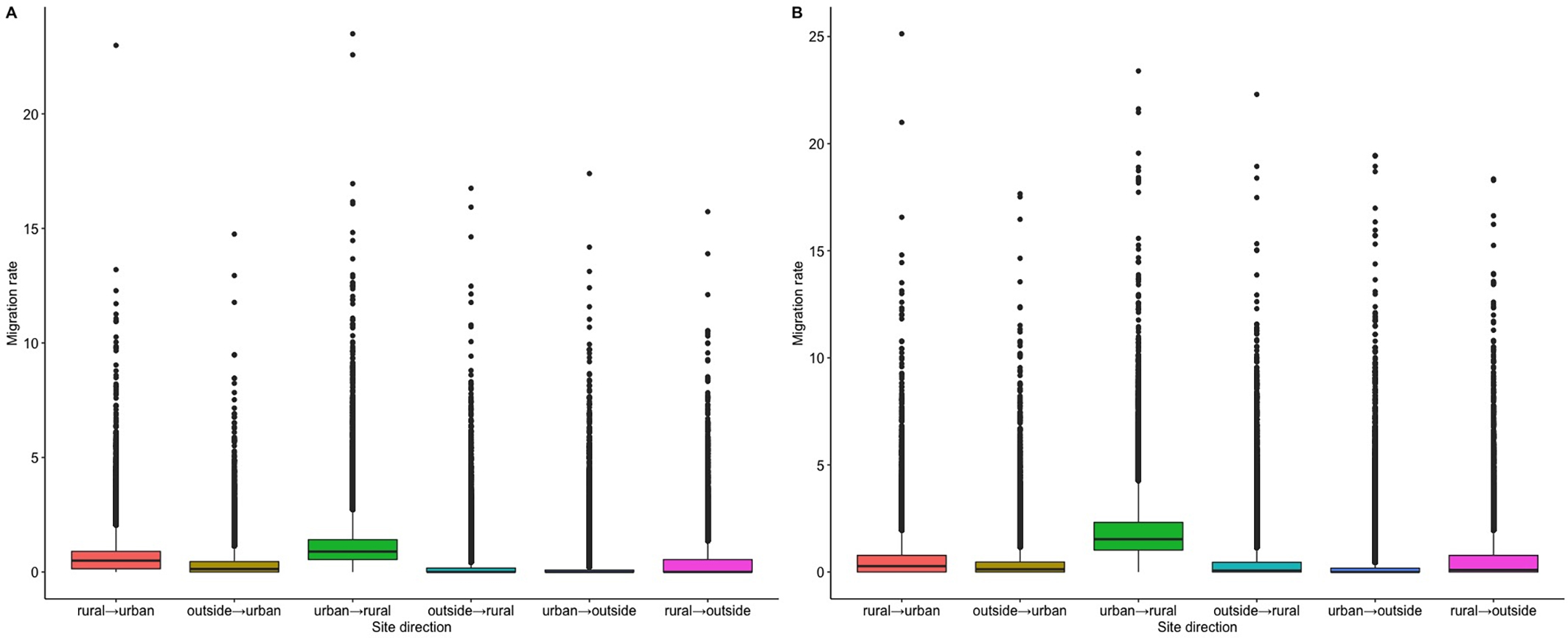

Supplement: 2 [file NIHMS1928423-supplement-2.jpg]

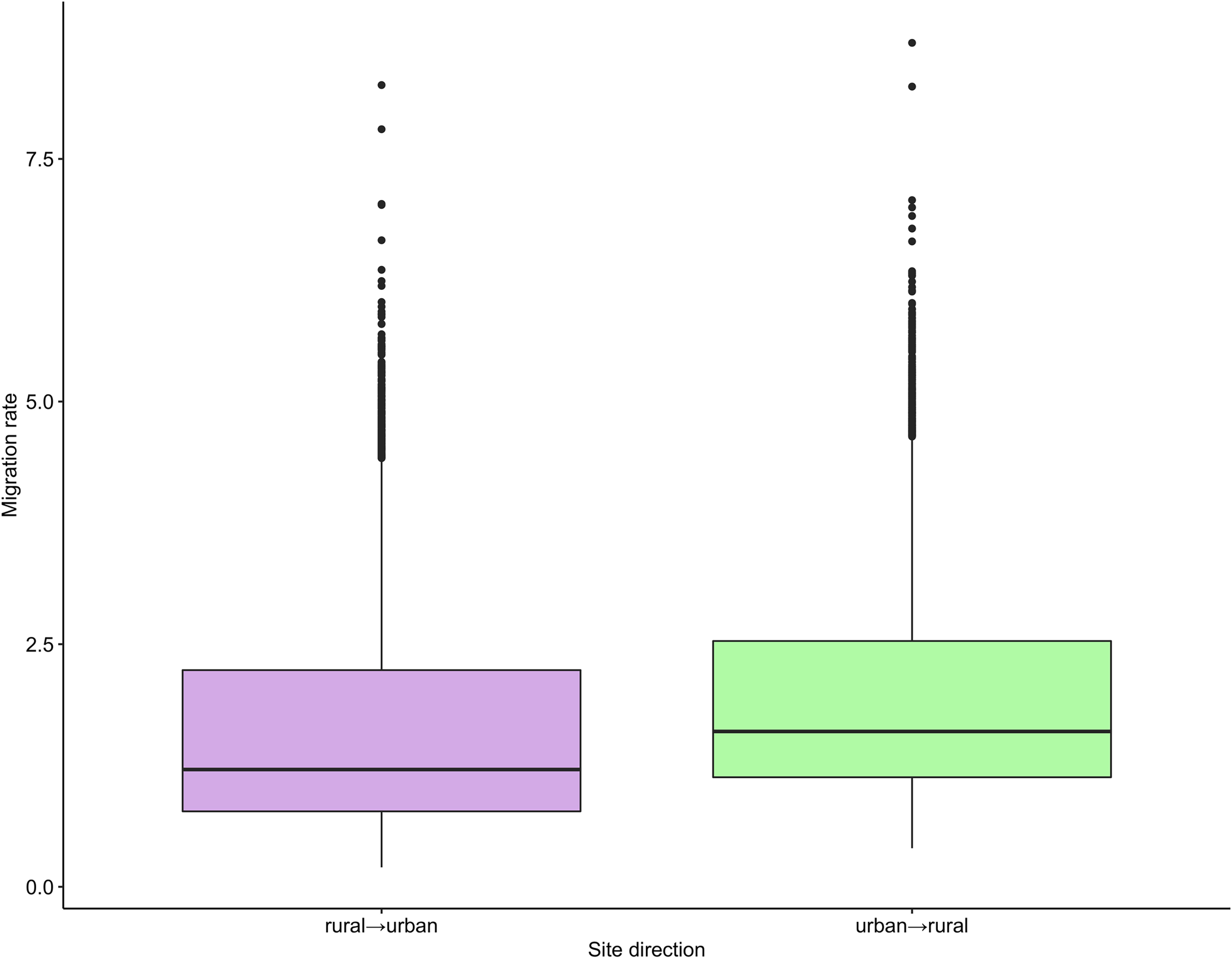

Supplement: 3 [file NIHMS1928423-supplement-3.jpg]

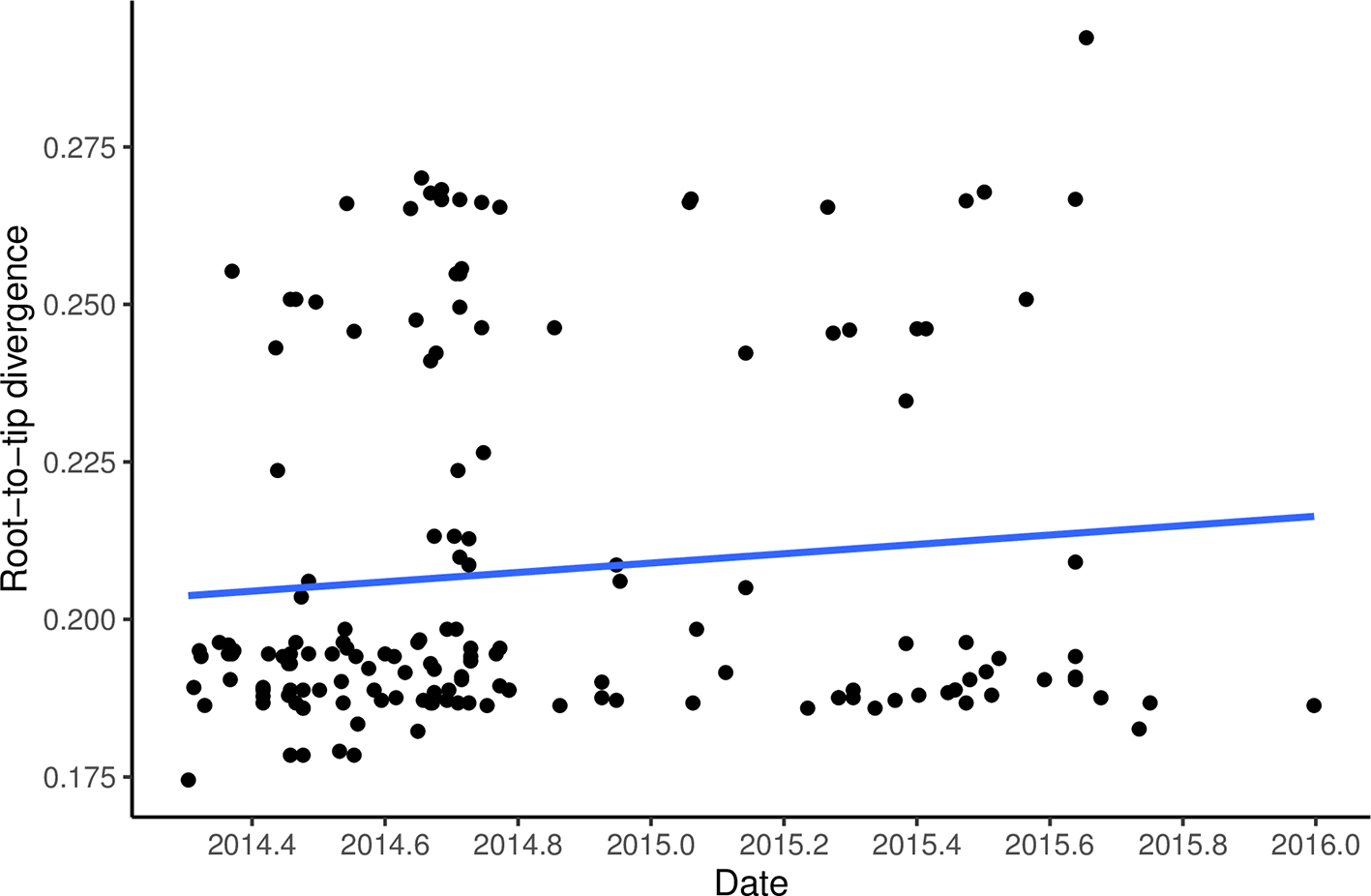

Supplement: 1 [file NIHMS1928423-supplement-1.jpg]
